# Neutrophil to high-density lipoprotein cholesterol ratio and prognosis in acute ischemic stroke: a systematic review and meta-analysis

**DOI:** 10.1186/s12883-026-05110-1

**Published:** 2026-06-27

**Authors:** Hongyu Lin, Qianqian Liu, Yanyan Liu, Huachun Huang, Zhijian Liang

**Affiliations:** Department of Neurology, The First Affiliated Hospital of Guangxi Medical University, Nanning, Guangxi Zhuang Autonomous Region China

**Keywords:** Neutrophil to high-density lipoprotein cholesterol ratio, Acute ischemic stroke, Prognosis, Adverse outcome, Meta-analysis

## Abstract

**Background:**

The neutrophil to high-density lipoprotein cholesterol ratio (NHR) is emerging as a potential composite biomarker integrating aspects of inflammation and lipid metabolism in the context of acute ischemic stroke (AIS). However, its clinical utility remains uncertain due to limited and inconsistent evidence across studies.

**Methods:**

A comprehensive systematic search was conducted in PubMed, Embase, and the Cochrane Library up to September 2025 to identify studies examining the association between NHR and adverse outcomes or mortality in AIS patients. Heterogeneity among studies was evaluated through sensitivity and subgroup analyses, and potential publication bias was explored using Egger’s test, recognizing the limited power due to the small number of included studies.

**Results:**

A meta-analysis including 4,138 patients from five studies suggested a possible association between higher NHR and adverse outcomes in AIS (OR 1.89, 95% CI: 1.18–3.02; I² = 82%), although substantial heterogeneity limits confidence in the pooled estimate. Sensitivity analyses yielded similar trends, but findings should be interpreted cautiously. Subgroup analyses indicated stronger associations for patients undergoing reperfusion therapy (OR 2.80, 95% CI: 1.79–4.38) and at 3-month follow-ups (OR 2.24, 95% CI: 1.51–3.32), while no significant associations were observed with conventional treatment or 1-month follow-ups. The pooled area under the curve (AUC) was 0.63, reflecting limited predictive performance. Only a single study examined mortality, reporting no significant association with NHR (OR 1.5; 95% CI: 0.47–4.74).

**Conclusions:**

Elevated NHR is associated with adverse outcomes in AIS, supporting its potential as a prognostic biomarker. However, evidence for mortality prediction is limited, and further prospective studies are needed.

**Supplementary Information:**

The online version contains supplementary material available at https://doi.org/10.1186/s12883-026-05110-1.

## Introduction

According to the World Stroke Organization Global Stroke Fact Sheet 2022, over 7.6 million new cases of ischemic stroke (IS) occur annually, with more than 77 million individuals currently living who have experienced IS. Annually, IS results in 3.3 million deaths and accounts for over 63 million years of healthy life lost due to stroke-related mortality and disability [1]. Consequently, ischemic stroke has emerged as a leading cause of death and disability globally [1]. Despite advancements in therapeutic interventions, such as intravenous thrombolysis and endovascular thrombectomy, a substantial proportion of patients do not achieve full recovery, highlighting the need for improved preventive and prognostic strategies informed by a more comprehensive understanding of stroke pathophysiology [2].

Atherosclerosis, characterized by low-grade, chronic inflammation of the arterial wall and the gradual accumulation of lipids and inflammatory cells [3–5], is a highly prevalent cause of IS [6]. Both inflammation and lipids metabolism are implicated in the initiation and progression of acute ischemic stroke (AIS). Post-ischemic inflammation is associated with blood-brain barrier (BBB) disruption, neuronal death, vasogenic edema, hemorrhagic transformation, and adverse neurological outcomes, based on both animal and human studies [7–13], although evidence remains limited. Among these pathological processes, neutrophils have been suggested to play a central role in AIS pathophysiology. Neutrophils are among the first immune cells to migrate into the brain tissue [7–9], where they release reactive oxygen species (ROS), elastases, and proteolytic enzymes such as matrix metalloproteinase-9 (MMP-9), potentially contributing to extracellular matrix degradation and further BBB compromise [13]. Conversely, circulating apolipoproteins are increasingly recognized as mediators at the interface of lipid metabolism, inflammation, endothelial dysfunction, and thrombosis [2]. High-density lipoprotein (HDL) in particular exhibits anti-inflammatory, anti-thrombotic, and antioxidant properties, alongside its role in reverse cholesterol transport [14–16]. HDL cholesterol (HDL-C) is commonly used to assess HDL levels and may partially reflect HDL functionality.

Recently, the neutrophil to high-density lipoprotein cholesterol ratio (NHR) has emerged as a potential composite biomarker integrating inflammatory and lipid metabolic pathways relevant to AIS. Clinical studies reported associations between NHR and the severity [17–19], early progression [20], adverse clinical outcomes [18, 21–23], and mortality [22] in AIS patients. However, findings are conflicting, and some studies have observed no significant correlation between NHR and adverse clinical outcomes [17, 24].

Given the limited and conflicting clinical evidence, the objective of this study is to systematically review the literature and conduct meta-analyses to explore the association between NHR and outcomes in AIS. These analyses are intended to provide preliminary insights rather than definitive conclusions, highlighting areas for future research.

## Materials and methods

This systematic review and meta-analysis were conducted in accordance with the guidelines outlined in the Preferred Reporting Items for Systematic Reviews and Meta-Analyses (PRISMA) statement [25]. It should be noted that the study protocol was not registered, which may introduce analytic flexibility and should be considered a limitation.

### Search strategy

We performed a systematic and comprehensive electronic search of PubMed, Embase, and the Cochrane Library up to September 3, 2025. The inclusion criteria were delineated using the PICO framework, including participants (P), intervention/exposure (I), comparison (C), and outcome (O). Given the limited literature in this domain, the search primarily focused on patients with acute ischemic stroke (P) and the neutrophil to high-density lipoprotein cholesterol ratio (I) to maximize retrieval. Search terms included ‘ischemic stroke,’ ‘neutrophil,’ ‘high-density lipoprotein cholesterol,’ and related variants. The detailed search strategy is provided in *Attachment 1*. No language or regional restrictions were applied, and references lists of relevant studies were screened to identify additional potentially eligible articles.

### Selection criteria

Studies were eligible if they met the following criteria: (1) prospective or retrospective observational studies (cohort, case-control, or cross-sectional studies); (2) populations diagnosed with AIS according to established criteria; (3) explicit definition of adverse outcomes, typically using the Modified Rankin Scale (mRS); (4) provision of odds ratios (OR) with 95% confidence intervals (CI) for the association between NHR and AIS outcomes or mortality, or provision of AUC and 95% CI for NHR in predicting adverse outcomes. Studies were excluded if they recruited only cases, were published as abstracts without full text, involved animal experiments, or were reviews, comments, or letters. In cases of duplicate data, the study with the largest sample or most comprehensive information was selected.

Two independent researchers (H.-Y. L. and Q.-Q. L.) screened titles and abstracts for eligibility after removing duplicate records, and another pair of researchers (Y.-Y. L. and H.-C.H.) independently assessed full texts. Disagreements were resolved by discussion, emphasizing consensus and minimizing selection bias.

### Definition of outcomes

The primary outcome was the association between the NHR and adverse outcomes, as defined using the mRS, in AIS patients. Secondary outcomes included the predictive performance of NHR for adverse outcomes (pooled AUC) and the association with mortality. Due to the small number of studies assessing mortality, this outcome is considered exploratory.

### Data extraction

Two researchers independently extracted using a standardized form and validated it collaboratively. Extracted data included author, year, country, study design, study duration, inclusion/exclusion criteria, blood sample timing, sample size, adverse outcomes definition, and OR/AUC with 95% CIs. The covariates included in adjusted ORs were summarized where available, recognizing that heterogeneity in adjustments may affect pooled estimates.

### Quality assessment

All included studies were retrospective cohort studies. Study quality was assessed using the Newcastle-Ottawa Scale (NOS) [26] for cohort studies, which evaluates methodological quality across three domains: selection of study groups, comparability of cohorts, and outcome assessment. NOS scores range from 0 to 9, with studies categorized as low quality (1–3), moderate quality (4–6), or high quality (7–9). Two investigators independently performed the quality assessment, and discrepancies were resolved through discussion.

### Statistical analysis

When adjusted ORs were reported, they were preferentially used; otherwise, univariate ORs were included. Heterogeneity was quantified using the I² statistic, with values ≥ 50% considered indicative of substantia heterogeneity. Random-effects models were applied when I² ≥50%, and fixed-effects models were applied when I² <50%, consistent with standard meta-analytic practice. Sensitivity analyses were conducted to evaluate the influence of individual studies. Subgroup analyses were performed to explore potential sources of heterogeneity, including treatment strategy (conventional therapy vs. reperfusion therapy) and endpoint timing (one month vs. three months). All subgroup findings should be considered preliminary and hypothesis-generating. Egger’s test was used to explore potential publication bias; however, with only five studies, this test is underpowered and its results should be interpreted cautiously.

The AUC for adverse outcome prediction was pooled as an exploratory estimate, acknowledging differences in thresholds, populations, and models across studies. Statistical significance was defined as *p* < 0.05. Meta-analyses were performed using Stata software, version 18.0 (StataCorp LP, College Station, TX, USA).

## Results

### Search results

**Fig. 1:**
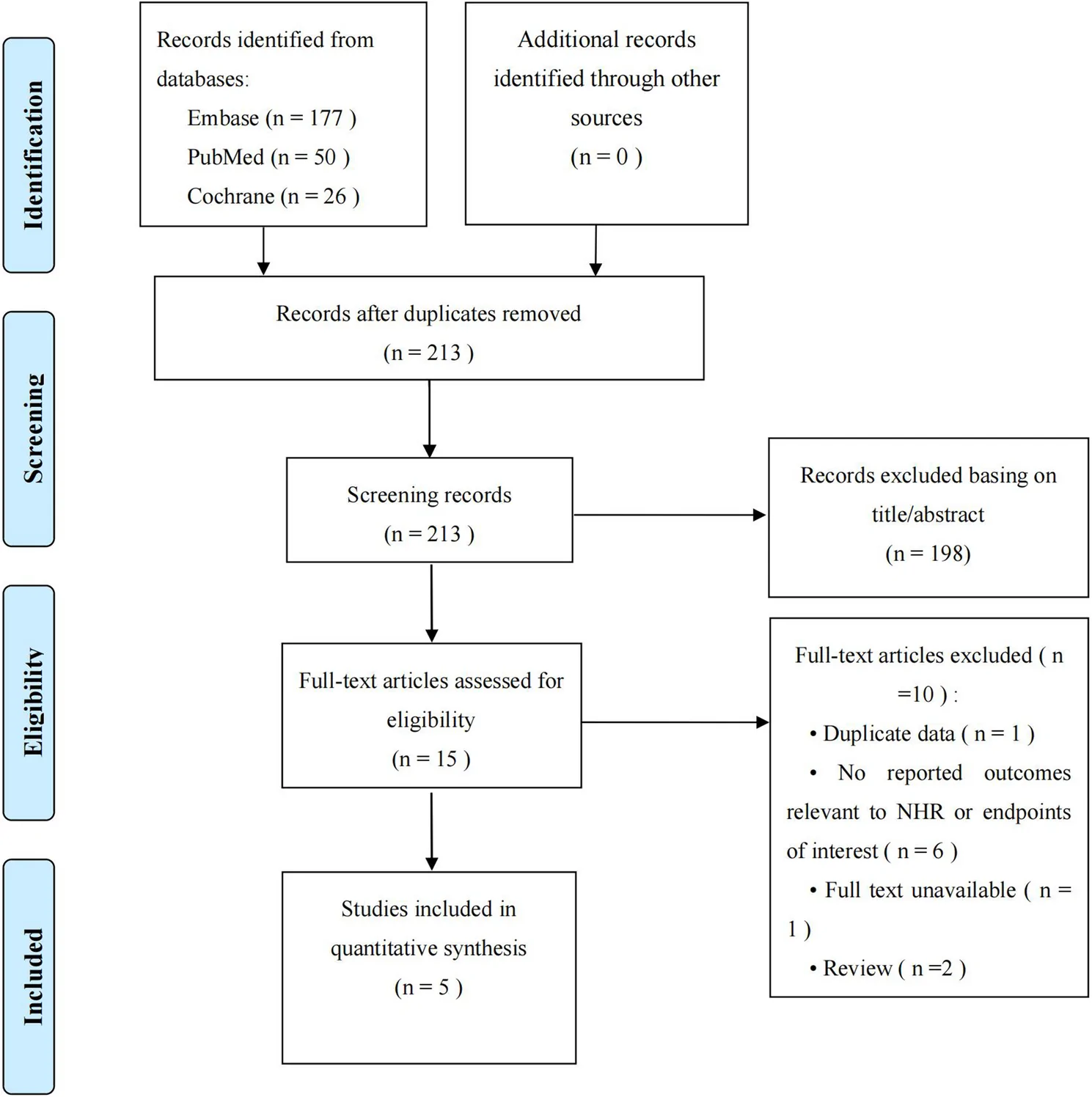
Flowchart of literature search and study selection for the meta-analysis. Legend: PRISMA flow diagram illustrating the systematic literature search and selection process for studies evaluating the association between neutrophil to high-density lipoprotein cholesterol ratio (NHR) and adverse outcomes in acute ischemic stroke (AIS). The diagram shows the number of records identified, screened, assessed for eligibility, and included in the final analysis, with reasons for exclusion at each stage

The literature search and selection process is illustrated in Fig. 1 using the PRISMA flow diagram. A total of 253 articles were identified through database searching (Embase = 177, PubMed = 50, and Cochrane = 26), and 40 duplicates were removed, leaving 213 articles for initial screening. After screening titles and abstracts, 15 articles were considered potentially eligible for full-text review. Of these, 10 studies were excluded for the following reasons: data duplication (*n* = 1), absence of reported outcomes relevant to NHR or the study endpoints of interest (*n* = 6), inaccessible full text (*n* = 1), and classification as reviews (*n* = 2). Notably, Zhu et al. published two studies based on the same hospital database [17, 24]; the study with the longer duration and the larger sample size was selected [24]. For the article with unavailable full text [27], attempts to contact the corresponding author and the journal’s editorial office were unsuccessful. Additionally, Wang et al. [28] categorized AIS patients into four groups based on neutrophil and HDL-C levels; however, specific NHR values were not reported, and thus the study was excluded. Ultimately, five studies met the inclusion criteria [18, 21–24]. Among them, Li et al. [23] used the training cohort to validate NHR associations; only the 496 patients from this cohort were included in the meta-analysis.

### Study characteristics

The characteristics of included studies are presented in Table 1. All studies were retrospective and conducted in China, limiting generalizability. Observation periods ranged from 2 to 7 years, with sample sizes between 160 and 2,828, totaling 4,138 participants. One study was multicenter [21]; the remaining were single-center studies [18, 22–24]. Two studies included patients receiving alteplase treatment [18, 23], one study included patients undergoing mechanical thrombectomy (MT) with complete recanalization [21], and two studies included patients receiving only conventional treatment [22, 24]. Blood samples were generally collected within 24 h, with Zhu et al. [24] collecting samples within one hour. Adverse outcomes definitions varied slightly across studies: Zhu et al. [24] used a 30-day endpoint versus 90 days in other studies, and mRS thresholds varied (≥ 2 versus > 2).

**Table 1:** Characteristics of the studies included in the meta-analysis

Study	Country	Design	Research time	Inclusion criteria	Main exclusion criteria	Blood specimen collection time	Sample size	Adverse outcome			Mortality (OR, 95%CI)
								Definition	OR (95%CI)	AUC (95%CI)	
Zhu et al 2024 [24]	China	retrospective study	September 2019 to February 2024	first stroke, AIS within 24 h of onset	received reperfusion therapy; severe cardiac, pulmonary, hepatic, renal, neoplastic or autoimmune disease or combined severe infection	within 1 hour of admission	306	mRS score >2 at 30 days	0.988 (0.792–1.234)†	NR	NR
Ci et al 2025 [21]	China	multicenter retrospective study	January 2016 and November 2023	received MT within 24 h and achieved complete recanalization with mTICI grade 3; occlusion of the internal carotid artery or M1/M2 segments of the middle cerebral artery	mRS score >2; severe infection, malignant tumor, or immune dysfunction; severe cardiac, hepatic, renal, or other major organ diseases	within 24 h after MT	348	mRS score >2 at 90 days	2.311 (1.248–4.278)†	0.592 (0.531–0.653)	NR
Chen et al 2020 [18]	China	retrospective study	2016 to 2018	treatment with intravenous rt-PA within 4.5 h of stroke onset	severe liver or kidney damage, rheumatic immune diseases, acute myocardial infarction, chronic inflammatory diseases, malignant tumor, or arteriovenous thrombosis of lower extremities	within 24 hours of admission	160	mRS score ≥2 at 3 months	4.570 (1.841–11.340)†	0.676 (0.598–0.748)	NR
Zhang et al 2024 [22]	China	retrospective study	January 2017 and July 2021	AIS within 48 h of onset	with inflammatory diseases or infectious disease; mRS score >2 before onset; received thrombolysis or other intervention	fasting for at least 8 h within 24 h of admission	2828	mRS score >2 at 90 days	1.69 (1.3–2.21)†	NR	1.5 (0.47–4.74)a
Li et al 2024 [23]	China	retrospective study	January 2016 and August 2022	treatment with intravenous rt-PA within 4.5 h of stroke onset	mRS score >2; received intravascular therapy; died within 24 h of receiving the thrombolysis;	after fasting in the morning following admission	496	mRS score >2 at 90 days	2.61 (1.04–6.51)†	NR	NR

*AIS* indicates acute ischemic stroke, *mRS *modified Rankin Scale, *mTICI *modified Thrombolysis in Cerebral Infarction, *OR *odd ratio, *CI *confidence interval, *AUC *area under curve, *MT *mechanical thrombectomy, *rt-PA *recombinant tissue plasminogen activators, *NR *not reported

†: Adjusted OR (95% CI)

### Risk of bias assessment

NOS scores of the five included studies ranged from 7 to 8 (Table 2), indicating high methodological quality. Most studies achieved full scores in the selection and comparability domains, with adequate control for major confounding factors such as age, baseline stroke severity, and vascular risk factors. Point deductions were uniformly observed in the outcome domain, as none of the studies provided explicit data on follow-up completeness or loss to follow-up rates. Two studies further lost one point each in the selection domain because they did not clearly verify that the primary outcome was absent at study baseline.

**Table 2:** Studies quality of included studies based on the Newcastle-Ottawa Scales

Study	Selection				Comparability	Outcome			Total Score
	Exposed Cohort	Nonexposed Cohort	Ascertainment of Exposure	Outcome of Interest		Assessment of Outcome	Length of Follow-up	Adequacy of Follow-up	
Zhu et al. 2024 [24]	*	*	*	*	**	*	*	-	8
Ci et al. 2025 [21]	*	*	*	*	**	*	*	-	8
Chen et al. 2020 [18]	*	*	*	-	**	*	*	-	7
Zhang et al. 2024 [22]	*	*	*	*	**	*	*	-	8
Li et al. 2024 [23]	*	*	*	-	**	*	*	-	7

Single asterisk (*) indicates 1 score, double asterisk (**) indicates 2 scores, and dash (-) indicates 0 scores

### Outcomes

#### Primary outcome: adverse outcome

**Fig. 2:**
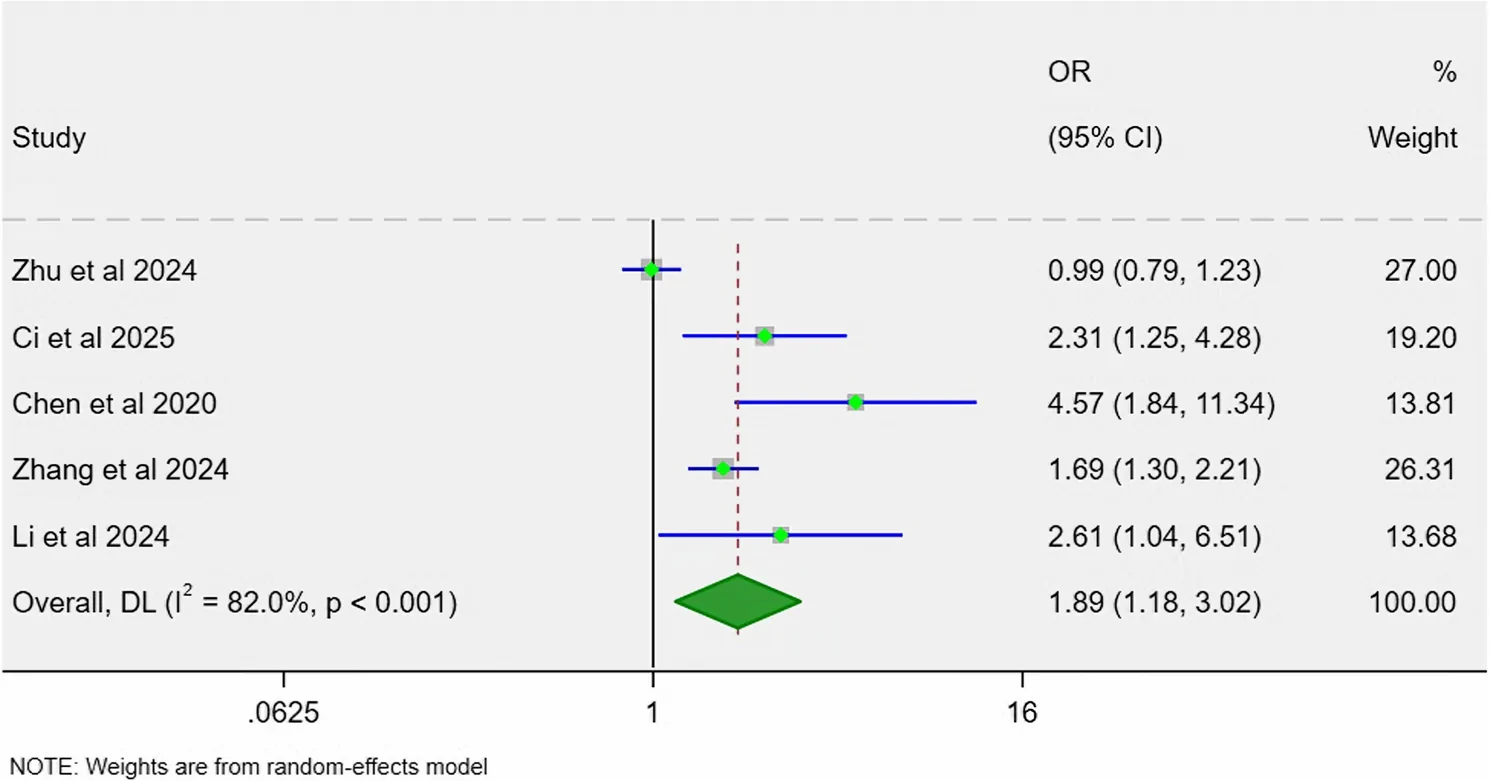
Forest plot of the association between NHR and adverse outcomes in AIS. Legend: Forest plot displaying the pooled odds ratios (ORs) and 95% confidence intervals (CIs) for the association between NHR and adverse outcomes in patients with acute ischemic stroke. The pooled estimate was calculated using a random-effects model due to significant heterogeneity (I² = 82%). Each study is represented by a square proportional to its weight, with horizontal lines indicating 95% CIs. The diamond represents the overall pooled effect estimate

**Fig. 3:**
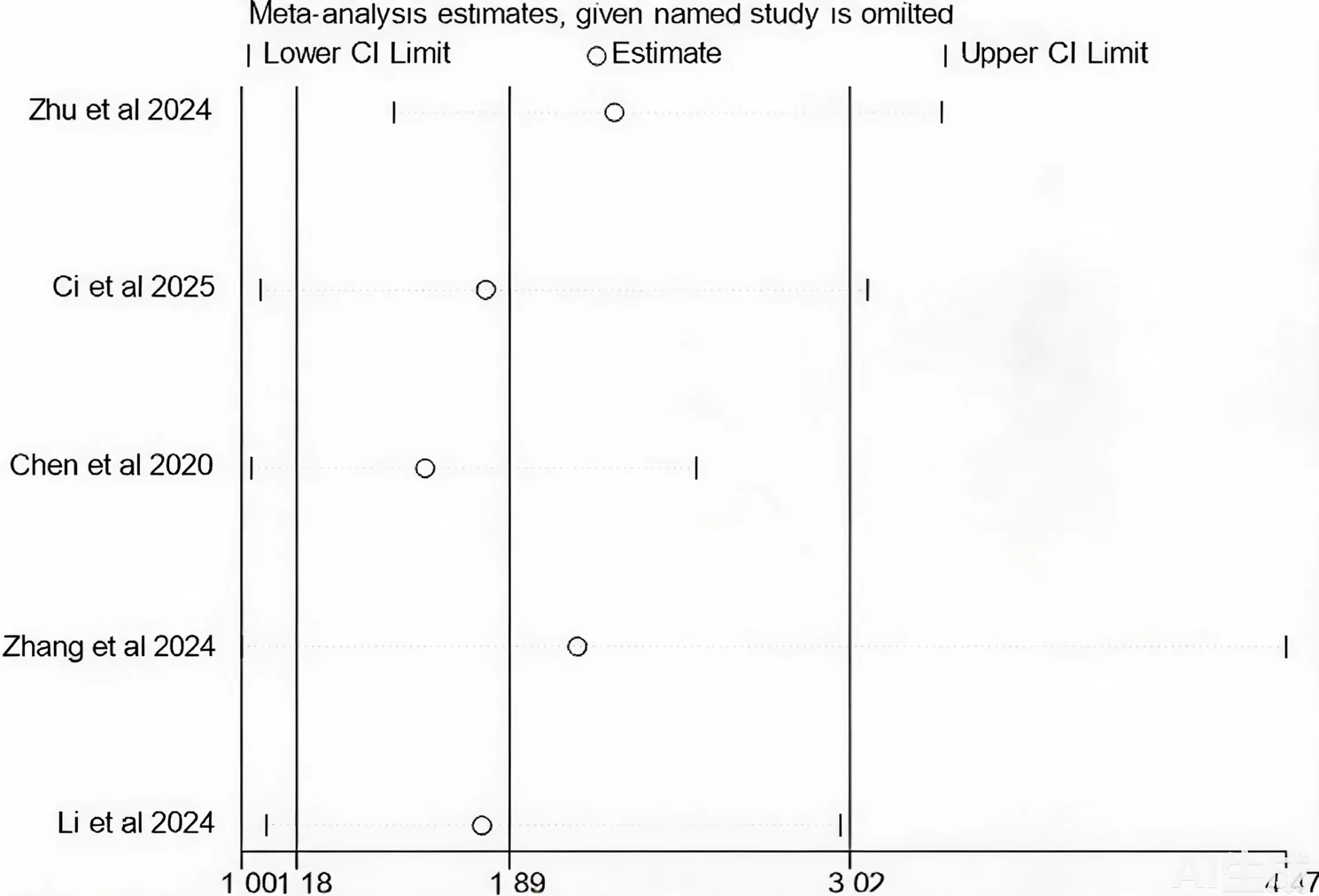
Sensitivity analysis for the association between NHR and adverse outcomes in AIS. Legend: Sensitivity analysis assessing the robustness of the pooled association between NHR and adverse outcomes in AIS. Each study was sequentially excluded to evaluate its impact on the overall pooled odds ratio. The analysis indicates that no single study substantially altered the pooled estimate

**Fig. 4:**
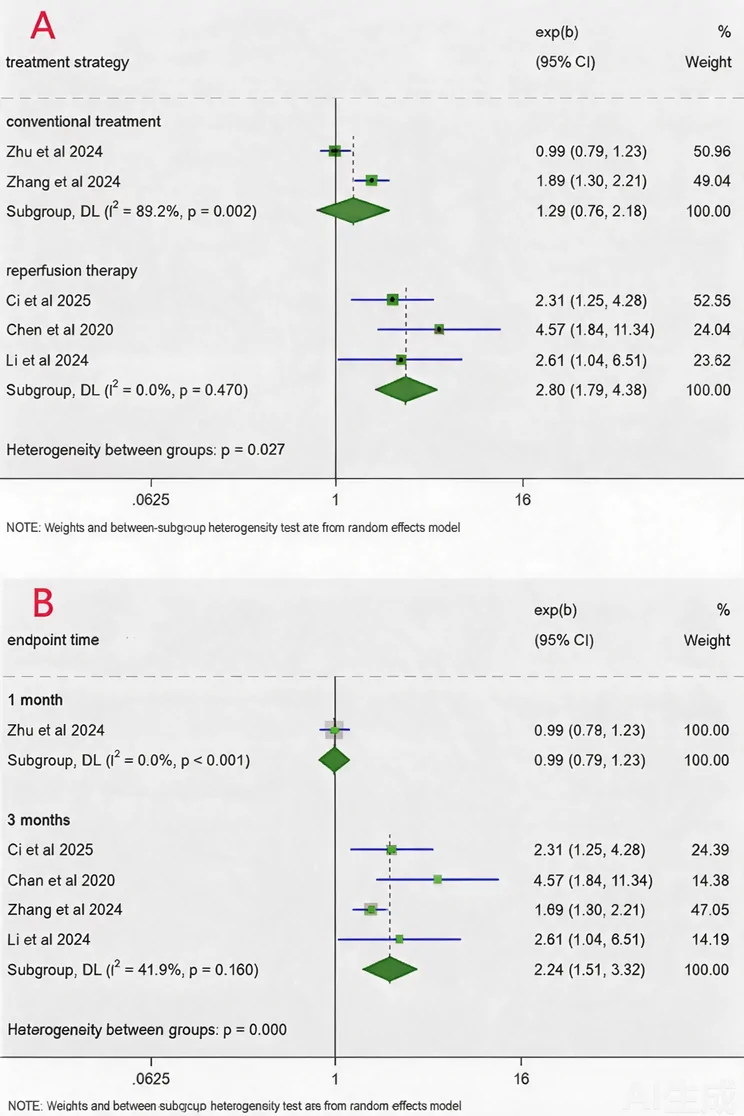
Subgroup analyses of NHR and adverse outcomes in AIS by treatment strategy and follow-up duration. Legend: Subgroup analyses showing pooled odds ratios (ORs) and 95% confidence intervals (CIs) for the association between NHR and adverse outcomes in AIS. Panel (**A**) presents results stratified by treatment strategy (reperfusion therapy vs conventional therapy). Panel (**B**) presents results stratified by endpoint timing (1-month vs 3-month follow-up). Subgroup estimates are exploratory due to the limited number of studies in some categories

The pooled OR for the association between the NHR and adverse outcomes was 1.89 (95% CI: 1.18–3.02; I² = 82%) (Fig. 2). Given the substantial heterogeneity, a random-effects model was employed for the meta-analysis. Sensitivity analyses suggested that exclusion of individual studies did not markedly alter pooled estimates (Fig. 3). Egger’s test indicated no apparent publication bias (*p* = 0.099), although the small number of studies limits interpretability (Attachment 2). Subgroup analyses, conducted for exploratory purposes, indicated that patients receiving reperfusion therapy showed an OR of 2.80 (95% CI: 1.79–4.38; I² = 0.0%), whereas those receiving conventional therapy showed an OR of 1.29 (95% CI: 0.76–2.18; I² = 89.2%) (Fig. 4A). Subgroup analyses by endpoint time suggested an OR of 2.24 (95% CI: 1.51–3.32; I² = 41.9%) for 3-month outcomes, while the single 1-month study yielded an OR of 0.99 (95% CI: 0.79–1.23; I² = 0%), precluding pooled meta-analysis (Fig. 4B). All subgroup results are preliminary and hypothesis-generating.

#### Secondary outcome: efficacy of NHR

**Fig. 5:**
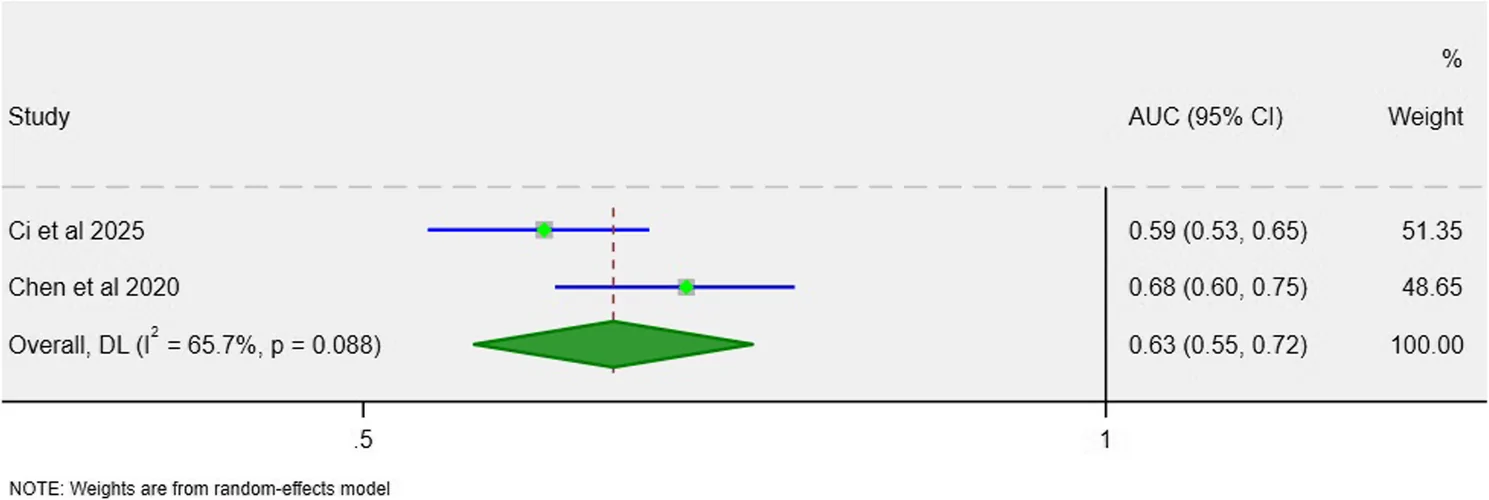
Forest plot of predictive performance of NHR for adverse outcomes in AIS. Legend: Forest plot displaying the pooled area under the curve (AUC) and 95% confidence intervals (CIs) for the ability of NHR to predict adverse outcomes in patients with AIS. The pooled estimate was calculated from two studies using a random-effects model, accounting for heterogeneity between studies (I² = 65.7%). The plot illustrates the predictive capacity of NHR, with the diamond representing the pooled AUC estimate

Two studies [18, 21] reported AUC values. The pooled AUC was 0.63 (95% CI: 0.55–0.72; I² = 65.7%) (Fig. 5). This result indicates limited predictive performance and should be interpreted cautiously due to heterogeneity and the small number of contributing studies.

#### Secondary outcome: mortality

Only Zhang et al. [22] reported mortality data(OR 1.5; 95% CI: 0.47–4.74). As the confidence interval includes 1.0, no statistically significant association was observed, and meta-analysis was not feasible. These findings should be considered exploratory given the single-study evidence.

## Discussion

### Main findings

The neutrophil to high-density lipoprotein cholesterol ratio (NHR) has emerged as a composite biomarker that integrates neutrophil-mediated pro-inflammatory activity with the anti-inflammatory and endothelial-protective functions of HDL-C. In this meta-analysis, higher NHR levels were associated with adverse outcomes in patients with acute ischemic stroke (AIS), demonstrating moderate predictive efficacy. However, current evidence is insufficient to draw firm conclusions regarding the relationship between NHR and mortality due to the limited number of studies.

### Clinical implications of NHR in prognosis

Emerging evidence indicates that NHR may serve as a practical prognostic biomarker in AIS, owing to its integration of inflammatory and lipid-related pathways. A systematic review by Gkantzios et al. highlighted the potential of NHR as a cost-effective, easily calculated, and broadly applicable marker for stroke prognosis [29]. Multiple studies have demonstrated that elevated NHR is associated with increased stroke severity, as assessed by the National Institutes of Health Stroke Scale (NIHSS), and correlates with early neurological deterioration [18, 30]. For example, Chen et al. reported significantly higher NHR levels in AIS patients with severe stroke compared to those with milder presentations (*p* = 0.011), and NHR positively correlated with NIHSS scores at admission, 24 h, and 7 days post-admission, with higher tertiles corresponding to worse neurological status [18].

In the AIS subtype of medial medullary infarction (MMI), Li et al. demonstrated that patients experiencing early neurological progression exhibited higher NHR values. Early progression was defined as an increase of ≥ 1 point in motor strength or ≥ 2 points in total NIHSS score within the first week. Receiver operating characteristic (ROC) analysis indicated that NHR significantly predicted early neurological progression in this cohort, with an AUC of 0.848 (*p* < 0.001) [30]. Additionally, retrospective studies have shown that elevated NHR at admission is independently associated with an increased risk of hemorrhagic transformation [31] and is a risk factor for decompressive craniectomy in AIS patients with large vessel occlusion undergoing mechanical thrombectomy [32]. Other well-established predictors of poor outcomes in AIS include NIHSS at admission [33], early neurological deterioration [34], hemorrhagic transformation [35], and need for decompressive surgery [36].

In this meta-analysis, subgroup analyses revealed that the association between NHR and adverse outcomes was stronger in patients receiving reperfusion therapy and at 3-month follow-up, while it was not observed in patients undergoing conventional treatment or at the 1-month endpoint. This may reflect differences in baseline stroke severity, infarct volume, or inflammatory response, or may be related to the limited number of studies and insufficient statistical power in these subgroups. Moreover, neurological recovery may continue beyond the first month, potentially masking early associations [37]. Collectively, these findings support the notion that NHR may provide complementary prognostic information in AIS, particularly in patients with more severe disease or receiving advanced therapies.

### Potential mechanistic considerations

The pathophysiological basis for the prognostic value of NHR in AIS likely involves the dynamic interplay between neutrophil-mediated inflammation and HDL-C–mediated vascular protection. Following ischemic injury, proinflammatory cytokines such as interleukin-6 (IL-6) and tumor necrosis factor-alpha (TNF-α) facilitate the recruitment of neutrophils to the ischemic brain tissue [10]. Neutrophil infiltration peaks at approximately 24 h and gradually declines over 7 days [8]. Neutrophils exacerbate cerebral injury by releasing reactive oxygen species (ROS), proteolytic enzymes (e.g., matrix metalloproteinases, elastase, proteinase 3), lipocalin-2, and forming neutrophil extracellular traps (NETs), which can compromise the blood-brain barrier, promote edema, and increase the risk of hemorrhagic transformation [7–11, 38].

HDL-C plays a central role in vascular protection. Its major components, including ApoA-I, ApoA-II, and ApoM, mediate reverse cholesterol transport, antioxidant activity, anti-inflammatory effects, anti-apoptotic signaling, and endothelial function [2]. ApoA-I additionally inhibits the hyperadhesive formation of von Willebrand factor, reducing platelet adhesion and thrombus formation under shear flow. ApoA-II contributes to HDL maturation and antioxidative properties, while ApoM facilitates cholesterol efflux and enhances endothelial nitric oxide production, reinforcing HDL’s vasculoprotective effects.

Reciprocal interactions between neutrophils and HDL-C further underscore the mechanistic relevance of NHR. Enzymes released during NET formation, such as myeloperoxidase, NADPH oxidase, and nitric oxide synthase, can modify HDL and reduce its protective function [39]. Low-density granulocytes, a specific subset of neutrophils identified in systemic lupus erythematosus, can interact with modified HDL to promote foam cell formation and contribute to the development of inflammatory high-risk plaques [40]. Conversely, HDL can inhibit neutrophil adhesion, migration, and cytokine production, mitigating inflammatory injury [41, 42]. ApoA-I and HDL have also been shown to reduce neutrophil adhesion to a platelet monolayer under shear flow and to attenuate neutrophil spreading and migration, while ApoA-I inhibited leukocyte recruitment to the endothelium in an acute in vivo model of inflammation [42].

Therefore, an elevated NHR may reflect either an intensified neutrophil-mediated inflammatory response or a diminished protective capacity of HDL-C, thereby explaining its association with adverse outcomes in AIS.

### Strengths, limitations, and future directions

This meta-analysis represents the first comprehensive synthesis of studies investigating the association between NHR and outcomes in AIS. Strengths of the present study include a systematic literature search conducted according to PRISMA guidelines, independent study selection and data extraction, quality assessment using the NOS, and the incorporation of sensitivity and subgroup analyses to explore potential sources of heterogeneity. In addition to pooled effect estimates, predictive-performance data were also summarized, providing a broader evaluation of the potential clinical relevance of NHR.

Nevertheless, several important limitations should be considered when interpreting the findings. First, all included studies were retrospective observational studies conducted in China, which substantially limits the external validity and generalizability of the results to other ethnic and healthcare populations. Furthermore, the study protocol was not preregistered, which may increase the possibility of analytic flexibility and selective reporting. Considerable heterogeneity was observed in the primary analysis (I² = 82%), likely reflecting both methodological and clinical differences among studies, including variations in treatment modalities, endpoint timing, blood sampling time, outcome definitions, and covariate adjustment strategies. Notably, adverse outcome definitions were not fully uniform across studies, with differences in follow-up duration (30-day versus 90-day outcomes) and mRS cutoff thresholds (mRS ≥ 2 versus > 2), which may have affected comparability of pooled estimates. In addition, blood samples were collected at different time points following admission, despite NHR being a potentially dynamic inflammatory marker, which may have contributed to interstudy variability. Another important limitation is that pooled analyses incorporated both adjusted and unadjusted ORs, while the covariates included in multivariable models differed substantially across studies; therefore, residual confounding cannot be excluded. Although the included studies generally achieved relatively high NOS scores, these assessments primarily reflect methodological quality rather than certainty of evidence. Given the retrospective observational design of all included studies and the limited number of eligible studies, the overall strength of evidence should still be interpreted cautiously. Publication bias could also not be reliably assessed because only five studies were available, rendering Egger’s test underpowered and of limited interpretability. The evidence regarding predictive performance and mortality was similarly limited. The pooled AUC analysis was based on only two studies and demonstrated moderate heterogeneity, while differences in thresholds, study populations, and predictive models may reduce the interpretability of pooled AUC estimates. Likewise, mortality analysis relied on a single study, precluding meaningful quantitative synthesis and limiting conclusions regarding the relationship between NHR and mortality. In addition, subgroup analyses should be considered exploratory and hypothesis-generating because several subgroup estimates were derived from only a small number of studies or single-study analyses. Finally, standardized NHR cutoff values have not yet been established, further limiting immediate clinical applicability.

Future prospective, multicenter studies with larger and more diverse populations are required to validate the prognostic value of NHR across different stroke subtypes, treatment strategies, and demographic settings. Standardized outcome definitions, uniform sampling protocols, and comprehensive adjustment for confounders will be essential to improve comparability across studies. Serial monitoring of NHR may provide additional insight into temporal inflammatory dynamics after AIS, while integration of NHR into multimodal prognostic models incorporating clinical, inflammatory, and metabolic parameters may improve risk stratification. Mechanistic studies are also warranted to clarify the biological interplay between neutrophils, HDL-C, and ischemic injury, as well as to establish validated cutoff values for future clinical application.

## Conclusions

Elevated NHR is associated with adverse outcomes in patients with AIS, supporting its potential as a prognostic biomarker that reflects systemic inflammation and lipid metabolism. However, the predictive value for mortality remains uncertain, and further prospective, multicenter studies are needed to validate its clinical applicability and establish standardized cutoff values.

## Supplementary Information

Supplementary Material 1.

Supplementary Material 2.

## Data Availability

All relevant data supporting this study’s conclusions are included within the manuscript or supplementary materials.

## Abbreviations

NHR: Neutrophil to high-density lipoprotein cholesterol ratio
IS: Ischemic stroke
AIS: Acute ischemic stroke
AUC: Area under curve
BBB: Blood-brain barrier
ROS: Reactive oxygen species
MMP: Matrix metalloproteinase
HDL: High-density lipoprotein
HDL-C: High-density lipoprotein cholesterol
PRISMA: Preferred Reporting Items for Systematic Review and Meta-Analysis
OR: Odds ratio
CI: Confidence interval
mRS: Modifed Rankin scale
NOS: Newcastle-Ottawa Scale
MT: Mechanical thrombectomy
NIHSS: National Institutes of Health Stroke Scale
MMI: Medial medullary infarction
NETs: Neutrophil extracellular traps
ApoA-I: Apolipoprotein A-I
ApoA-II: Apolipoprotein A-II
ApoM: Apolipoprotein M
vWF: Von Willebrand factor
IL-6: Interleukin-6
TNF-α: Tumor necrosis factor-alpha
IL-1β: Interleukin-1β
